# Evaluation of Recombinant Human FGF-2 and PDGF-BB in Periodontal Regeneration: A Systematic Review and Meta-Analysis

**DOI:** 10.1038/s41598-017-00113-y

**Published:** 2017-03-06

**Authors:** Feifei Li, Fanyuan Yu, Xin Xu, Chunjie Li, Dingming Huang, Xuedong Zhou, Ling Ye, Liwei Zheng

**Affiliations:** 1 State Key Laboratory of Oral Diseases, National Clinical Research Center for Oral Diseases, West China Hospital of Stomatology, Sichuan University, Chengdu, China; 2 West China School of Stomatology, Sichuan University, Chengdu, China

## Abstract

The prognosis for successful treatment of periodontal diseases is generally poor. Current therapeutic strategies often fail to regenerate infected periodontium. Recently an alternative strategy has been developed that combines conventional treatment with the application of recombinant human growth factors (rhGFs). But ambiguities in existed studies on the clinical efficacy of rhGFs do not permit either the identification of the specific growth factors effective for therapeutic interventions or the optimal concentration of them. Neither is it known whether the same rhGF can stimulate regeneration of both soft tissue and bone, or whether different patient populations call for differential use of the growth factors. In order to explore these issues, a meta-analysis was carried out. Particular attention was given to the therapeutic impact of fibroblast growth factor 2(FGF-2) and platelet derived growth factor BB (PDGF-BB). Our findings indicate that 0.3% rhFGF-2 and 0.3 mg/ml rhPDGF-BB show a greater capacity for periodontal regeneration than other concentrations and superiority to control groups with statistical significance. In the case of patients suffering only from gingival recession, however, the application of rhPDGF-BB produces no significant regenerative advantage. The findings of this study can potentially endow clinicians with guidelines for the appropriate application of these two rhGFs.

## Introduction

Periodontal diseases, including gingival diseases and periodontitis, are a set of inflammatory diseases affecting the periodontium. They are often associated with progressive periodontal hard and soft tissue loss. The clinical outcome to such conditions is often the extraction of the affected teeth^1–3^. The alternative prognosis for teeth with severe alveolar bone loss or gingival recession is usually poor^1, 4^. Current periodontal therapeutic strategies, such as scaling and debridement, open flap surgery or guided tissue regeneration generally fail to achieve the regeneration of periodontium^4–7^. This limitation continues to be a clinical challenge and a major concern for periodontists. Fortunately, there is increasing evidence concerning the possible efficacy of certain additional interventions that may promote periodontal tissue renewal. Numerous experiments with animals as well as clinical trials indicate that the application of GFs, such as PDGF-BB, FGF-2 and others, is capable of stimulating the regeneration of periodontium and may be a promising forward step in the evolution of regenerative periodontology^8–14^.

Platelet-derived growth factors (PDGF) are now known to be actively involved in tissue regeneration and wound healing^10^_._ Research into the ability of PDGFs to promote periodontal tissue regeneration was pioneered by Lynch in 1989^11^. Further studies revealed that PDGFs are released by blood platelets and, in response to injuries, bind specific cell surface receptors that promote the healing of wounds via chemotaxis and mitogenesis^15, 16^. At present, three different forms of PDGFs have been identified: PDGF-AA, PDGF-AB, and PDGF-BB. Among these, the efficacy of PDGF-BB in both soft and hard tissue regeneration has been most clearly demonstrated. It has been approved by the FDA for use in periodontal therapy in cases of intrabony defects, furcation lesions, and gingival recession^17^. Fibroblast growth factors (FGF) are another large family of growth factors that are actively involved in angiogenesis, wound healing and tissue regeneration. Among these, FGF-2 has been the most extensively studied^18^. With its ability to bind to heparin, FGF-2 displays broad mitogenic and angiogenic properties^19^. In some instances, it has been found to promote bone formation through accelerating the differentiation of osteoprogenitor cells. It has also been found to stimulate the proliferation and migration of periodontal ligament cells, which makes it a promising candidate for regenerating periodontal soft and hard tissue^8, 9, 20, 21^.

Up to date, as many as 17 randomized control trials (RCTs) have been carried out and published in English to evaluate the efficacy of rhPDGF-BB and rhFGF-2 in the treatment of periodontal defects^13, 14, 22–36^, However, only 12 studies meet our inclusion criteria. The substantial heterogeneity of these RCTs, however, makes it difficult for clinicians to determine either the actual level of efficacy of GFs or the specific concentrations which should be used. Taking RCTs on rhPDGF-BB as an example, studies from Nevins^33^ concluded that rhPDGF-BB was effective in the treatment of periodontal osseous defects^33^. In contrast, similar trials reported in Mishra^22^ showed no statistically significant differences in periodontal clinical parameters between the rhPDGF-BB treatment group and the control group^22^. Furthermore, the criteria for selection of patients for inclusion differed from one study to another. Some included patients with periodontal osseous defects^22, 27, 28, 33, 36^ while others selected a sample of patients who suffered from gingival recession^23, 25, 29^. As for research into rhFGF-2, as many as five distinct doses were used in four of the RCTs, creating clinical ambiguity concerning the most effective concentration of rhFGF-2^24, 30–32^. Such ambiguities created the need for a critical review to develop quantifiable evidence-based guidelines for clinical utilization.

In this light, two earlier meta-analyses had already focused on the effect of growth factors on periodontal repair^37, 38^. Unfortunately, each of these studies suffered from limitations that weakened their clinical value (The problems will be identified and discussed below). Subsequent to these studies, several later RCTs were reported^24, 28, 32^, which significantly enlarged the overall sample size for our meta-analysis. This permitted us to independently evaluate the clinical efficacy of rhFGF-2 and rhPDGF-BB with different specific concentrations in periodontal repair. We focused on the following issues. Firstly, we attempted to ascertain whether rhPDGF-BB and rhFGF-2 can be deemed effective in regeneration of periodontium among patients suffering from either osseous defect or gingival recession. Secondly, we enquired whether the impact of periodontal therapy based on rhPDGF-BB and rhFGF-2 is affected by the concentration. Thirdly, we considered it important to identify whether periodontal hard tissue and soft tissue manifest different regeneration effects when treated with the same growth factors.

We furthermore reviewed the impact of other recombinant human growth factors, such as recombinant human growth differentiation factor-5 (rhGDF-5) and recombinant human insulin growth factor-1 (rhIGF-1) that had already been subjected to clinical experimentation regarding their impact on periodontal regeneration^26, 39, 40^, Because of the limited number of published studies about above mentioned factors, it is currently impossible to give a reliable quantitative evaluation of their effect. We will therefore briefly list their relevant results but will append the caveat that more clinical trials are required to reliably document their effectiveness in periodontal treatment.

## Results

### Search Results

In accordance with the inclusion and exclusion criteria described in methods section, twelve RCTs were eventually included in this meta-analysis.

### Characteristics of Included Studies

Twelve RCTs were included in this meta-analysis, and six of which were not included in previous meta-analysis. Four of the RCTs evaluated the efficacy of rhFGF-2 in treating periodontal infrabony defects (Cochran^24^, Kitamura^30^, Kitamura^31^ and Kitamura^32^), while the rest evaluated the efficacy of rhPDGF-BB on either periodontal infrabony defects (Mishra^22^, Jayakumar^27^, Maroo and Murthy^28^, Nevins^33^, Thakare^36^) or gingival recession (Carney^23^, Deshpnade^25^, McGuire^29^). In the four RCTs that dealt with the efficacy of rhFGF-2, a total of 5 different concentrations were utilized to assess their differential impact on several dependent variables, including bone fill percentage (BF%), linear bone growth(LBG) and gains in clinical attach levels (CAL-G). Among the 5 different concentrations, there was only 0.3% rhFGF-2 used in every RCTs. With the exception of one of the studies (Cochran^24^), the remaining RCTs used 3% hydroxypropylcellulose (HPC) as the carrier of rhFGF-2 and set it as the control.

As indicated above, five of the RCTs evaluated the impact of rhPDGF-BB on the treatment of periodontal osseous defects. All of these studies applied 0.3 mg/ml rhPDGF-BB to the intervention group with a view to exploring its impact on BF%, LBG, CAL-G, probing depth reduction (PDR) and gingival recession (GR). For the control groups, one of the studies (Mishra 2014) applied modified minimally invasive surgical technique (M-MIST). The remaining four studies utilized β-TCP as the carrier of rhPDGF-BB and also the control intervention. Three other studies examined the use of rhPDGF-BB on patients with gingival recession, specifically analyzing its impact on CAL-G, PRD, GR, width of keratinized gingiva (WKT), and root coverage percentage (RCP). It should be noted that they differ from each other with respect not only to the carrier of the rhPDGF-BB but also to the control interventions. In two of the studies (Deshpnade^25^ and McGuire^29^), the carrier was β-TCP, while the third study (Carney^23^) utilized acellular dermal matrix (ADM) as the carrier. There were differences in the control interventions as well. The control group of one study (Deshpnade^25^) received a sub-epithelial connective tissue graft, whereas the control group of the other two (Carney^23^ and McGuire^29^) received ADM or coronally positioned flap surgery. (Shown in Table 1).

**Table 1:** Characteristics of included studies.

STUDY ID	PATIENT				ARMS					F/U PERIOD (MONTHS)	OUTCOMES	STUDY TYPE (STUDY DESIGN)
	NUMBER	AGE (YEAR)	GENDER (F/M)	DEFECT TYPE	INTERVENTION			CONTROL				
					GROWTH FACTOR	CARRIER	NUMBER	ITEM	NUMBER			
Cochran^24^	88	^*^	34/54	OD	FGF2:0.1%	β-TCP	21	β-TCP	22	6	BF%, LBG, CAL-G	RCT, parallel
					FGF2:0.3%		22					
					FGF2:0.4%		23					
Kitamura^30^	80	^*^	49/31	OD	FGF2:0.03%	3%HPC	20	3%HPC	20	9	BF%, LBG, CAL-G	RCT, parallel
					FGF2:0.1%		20					
					FGF2:0.3%		20					
Kitamura^31^	267	^*^	141/126	OD	FGF2:0.2%	3%HPC	70	3%HPC	67	9	BF%, CAL-G	RCT, parallel
					FGF2:0.3%		65					
					FGF2:0.4%		65					
Kitamura^32^ STUDY A	328	^*^	200/128	OD	FGF2:0.3%	3%HPC	220	3%HPC	108	9	BF%, CAL-G	RCT, parallel
Kitamura^32^ STUDY B	158	^*^	98/60	OD	FGF2:0.3%	3%HPC	115	3%HPC	43	9	BF%, LBG, CAL-G	RCT, parallel
Mishra^22^	24	—	12/12	OD	PDGF-BB: 0.3 mg/ml	—	12	M-MIST	12	6	BF%,LBG,CAL-G, PRD, GR	RCT, parallel
Jayakumar^27^	54	25–75	29/25	OD	PDGF-BB: 0.3 mg/ml	β-TCP	27	β-TCP	27	6	BF%,LBG,CAL-G, PRD, GR	RCT, parallel
Maroo^28^	15	38.4 ± 7.6	—	OD	PDGF-BB: 0.3 mg/ml	β-TCP	15	β-TCP	15	9	BF%,LBG,CAL-G, PRD, GR	RCT, split-mouth design
Thakare^36^	18	35.76 ± 7.38	—	OD	PDGF-BB: 0.3 mg/ml	β-TCP	9	β-TCP+HA	9	12	BF%,LBG,CAL-G, PRD, GR	RCT, split-mouth design
Nevins^33^	180	25–75	72/108	OD	PDGF-BB: 0.3 mg/ml PDGF-BB: 1 mg/ml	β-TCP	60 61	β-TCP	59	6	BF%,LBG,CAL-G,	RCT, parallel
Carney^23^	17	30–69	12/5	GR	PDGF-BB: 0.3 mg/ml	ADM	20	ADM	20	6	CAL-G, PRD, GR, WKT	RCT, split-mouth design
Deshpande^25^	36	26.9 ± 5.5	—	GR	PDGF-BB: 0.3 mg/ml	β-TCP	12	CPF	12	6	CAL-G, PRD, GR, WKT, RCP	RCT, parallel
McGuire^29^	30	43.8 ± 10.7	—	GR	PDGF-BB:0.3 mg/ml	β-TCP	30	CTG	30	6	CAL-G, PRD, GR, WKT, RCP	RCT, split-mouth design

*Age range difffered in each group (raw data shown in original paper); —: no information; F/M: female number versus male number; F/U, Follow-up; OD: osseous defect; GR: gingival recession; PDGF: platelet-derived growth factor; FGF2: Fibroblast growth factor 2; HPC: Hydroxypropylcellulose; M-MIST: modified minimally invasive surgical technique; ADM: acellular dermal matrix; CPF: coronally positioned flap; CTG: subepithelial connective tissue graft; BF%: percentage of bone fill; LBG: linear bone growth; CAL-G: clinical attachment level regained; PRD: probing depth reduction; GR:gingival recession; WKT: width of keratinized gingiva; RCP: percentage of root coverage.

### The Effect of Rhfgf-2 on Periodontal Regeneration

#### Primary outcomes

**Figure 1:**
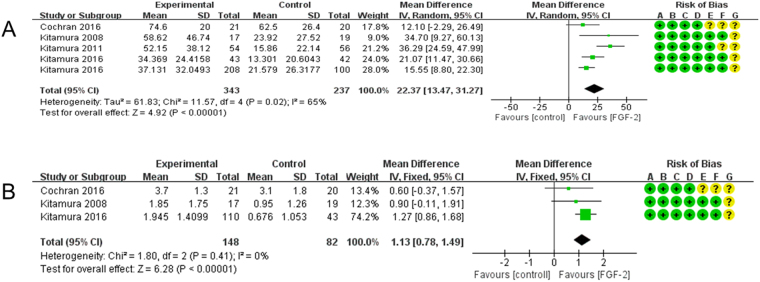
(**A**) Forest plot of comparison: 0.3% FGF2 groups was compared with control groups among patients with osseous defects, outcome: BF%. (**B**) Forest plot of comparison: 0.3% FGF2 groups was compared with control groups among patients with osseous defects, outcome: LBG. Risk of bias legends: (A) Random sequence generation (selection bias); (B) Allocation concealment (selection bias); (C) Blinding of particepants and personnel (performance bias); (D) Blinding of outcome assessment (Detection bias); (E) Incomplete outcome data (attrition bias); (F) Selective reporting (reporting bias) (G) Other bias.

To analyze the impact of 0.3% rhFGF-2 on the BF% and on the LBG, data from four of the RCTs (Cochran^24^, Kitamura^30^, Kitamura^31^ and Kitamura^32^) were pooled and analyzed together (Fig. 1). Cochran^24^ reported a BF% of 74.6 ± 20.0 (N = 21) in the 0.3% rhFGF-2 group and 62.5 ± 26.4 (N = 20) in the control group at the follow-up endpoint. Kitamura^30^ and Kitamura^31^ in contrast showed 58.62 ± 46.74 (N = 17) and 52.15 ± 38.12 (N = 54) in their respective intervention groups. Their control groups yielded 23.92 ± 27.52 (N = 19) and 15.86 ± 22.14 (N = 56) respectively. In Kitamura^32^, it reported two independent RCTs (RCT A and RCT B), and it showed that the BF% for the intervention groups was 34.369 ± 24.4158 (N = 43) and 37.131 ± 32.0493 (N = 208) respectively. However, in the two control groups it was 13.301 ± 20.6043 (N = 42) and 21.579 ± 26.3177 (N = 100). For our meta-analysis we pooled the data and found that the BF% in the 0.3% rhFGF-2 intervention groups was 22.37% higher, constituting a statistically significant difference (95%CI = 13.47~31.27, *p* < 0.00001) from the BF% in the control groups (Fig. 1A). In addition, meta-analysis of LBG also yielded results favorable to intervention groups using 0.3% rhFGF-2 with low heterogeneity (MD = 1.13, 95%CI = 0.78~1.79, *p* < 0.00001, χ^2^ = 1.80, *p* = 0.41, I^2^ = 0%; Fig. 1B).

**Figure 2:**
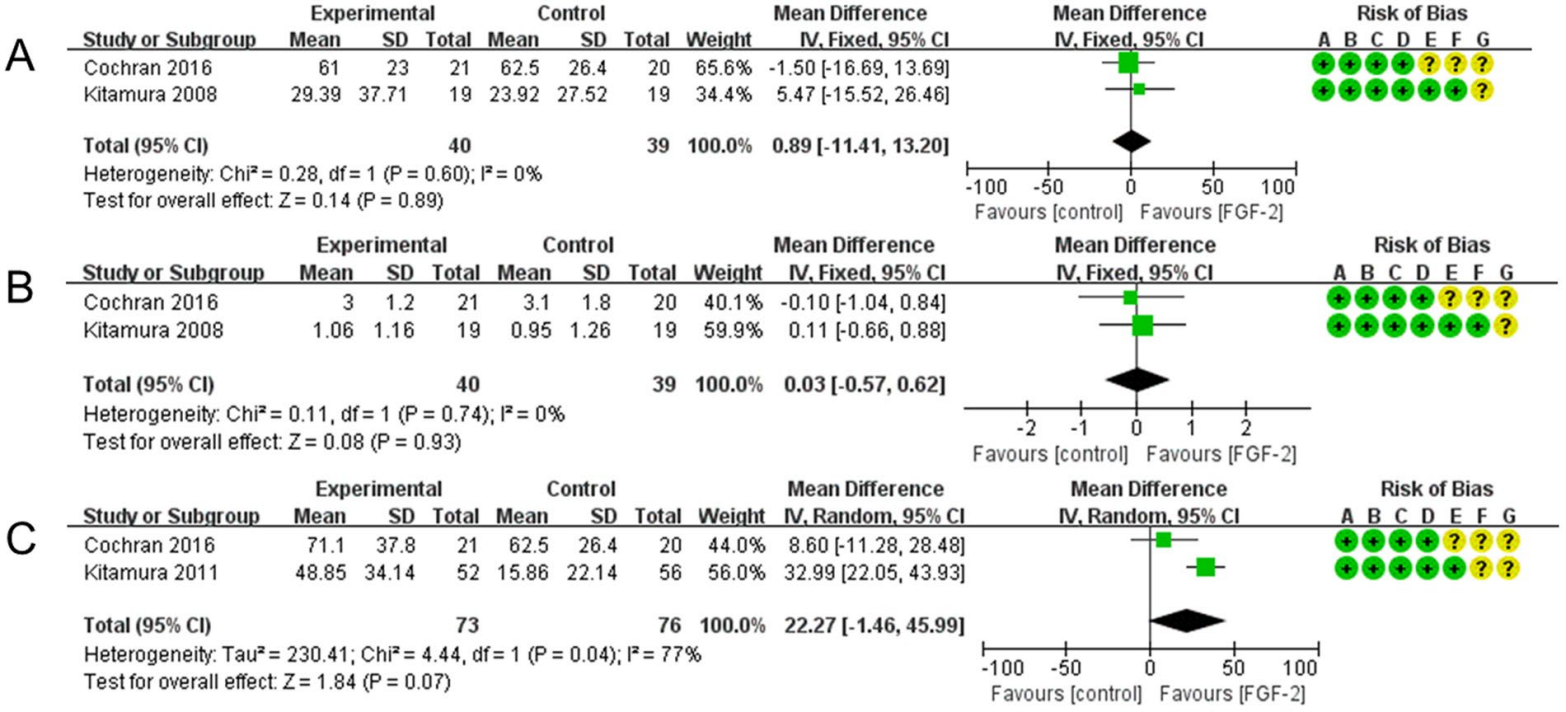
(**A**) Forest plot of comparison: 0.1% FGF2 groups was compared with control groups among patients with osseous defects, outcome: BF%. (**B**) Forest plot of comparison: 0.1% FGF2 groups was compared with control groups among patients with osseous defects, outcome: LBG. (**C**) Forest plot of comparison: 0.4% FGF2 groups was compared with control groups among patients with osseous defects, outcome: BF%.

In addition, statistical analysis was carried out on the clinical efficacy of 0.1% and 0.4% rhFGF-2. The objective was to determine whether the effect of periodontal therapy utilizing rhFGF-2 was concentration dependent. The results for the intervention groups yielded higher figures for both dosages than those found in the control groups but the differences were not statistically significant (Fig. 2). In exact figures, the lower-dosage patients (0.1% rhFGF-2) were found to achieve only 0.89% higher BF% than the patients in the control groups (95%CI = −11.41~13.20, *p* = 0.89; Fig. 2A). They furthermore had 0.03 mm more LBG than the control groups (95%CI = −0.57~0.62, *p* = 0.93; Fig. 2B). The higher dosage groups (0.4% rhFGF-2) fared somewhat better, with 22.27% higher BF% compared to the controls (Fig. 2C). But even these differences did not reach the 0.05 level of significance (95%CI = −1.46~45.99, *p* = 0.07).

### Secondary outcomes

The figure referred to as CAL-G measures the level of repair of periodontal hard and soft tissue. Four of the above-mentioned RCTs (Cochran^24^, Kitamura^30^, Kitamura^31^ and Kitamura^32^) explored the effect of 0.3% rhFGF-2 on CAL-G (S1. B). The CAL-G achieved by the 0.3% rhFGF-2 treatment group, as reported by Cochran^24^, was 3 ± 1.4 (N = 21). The value for the control group was very close: 2.9 ± 2.1 (N = 20). Kitamura^30^ and Kitamura^31^ presented similarly close figures for treatment and control patients. The CAL-G measure for treatment patients in the two studies was 2.18 ± 1.33 (N = 17) and 2.35 ± 1.78 (N = 55) respectively. The control group figures were 2.63 ± 1.54 (N = 19) and 2.12 ± 1.72 (N = 57) respectively. In one of the two independent RCTs reported by Kitamura^32^ (RCT A), the CAL-G value for the treatment group was 2.1 ± 1.58 (N = 213). The results for the control group were almost identical: 2 ± 1.48 (N = 106). In another RCT reported in Kitamura^32^ (RCT B) the treatment group that received the 0.3% rhFGF-2 treatment achieved a CAL-G value of 2.7 ± 1.29 (N = 110), larger than the figure for the control group: 1.7 ± 1.39 (N = 110). Our meta-analysis combined all these results and revealed that the treatment group regained more CAL than the control group, but not a level of statistical significance (MD = 0.27, 95%CI = −0.26~0.81, *p* = 0.31; S1. B). Meanwhile the meta-analysis of the effects of using a lower concentration of rhFGF-2(0.1%) indicated that the intervention group actually achieved a level of CAL-G that was −0.52 mm lower than that of the control group (The difference is not statistically significant; S1. A). The CAL-G figure achieved by those treated with the higher concentration of rhFGF-2 (0.4%) was 0.43 mm higher than that of the control group. The difference between them, however, was not statistically significant (S1. C).

### The Effect of Rhpdgf-Bb on Patients with Osseous Defect

#### Primary outcomes

**Figure 3:**
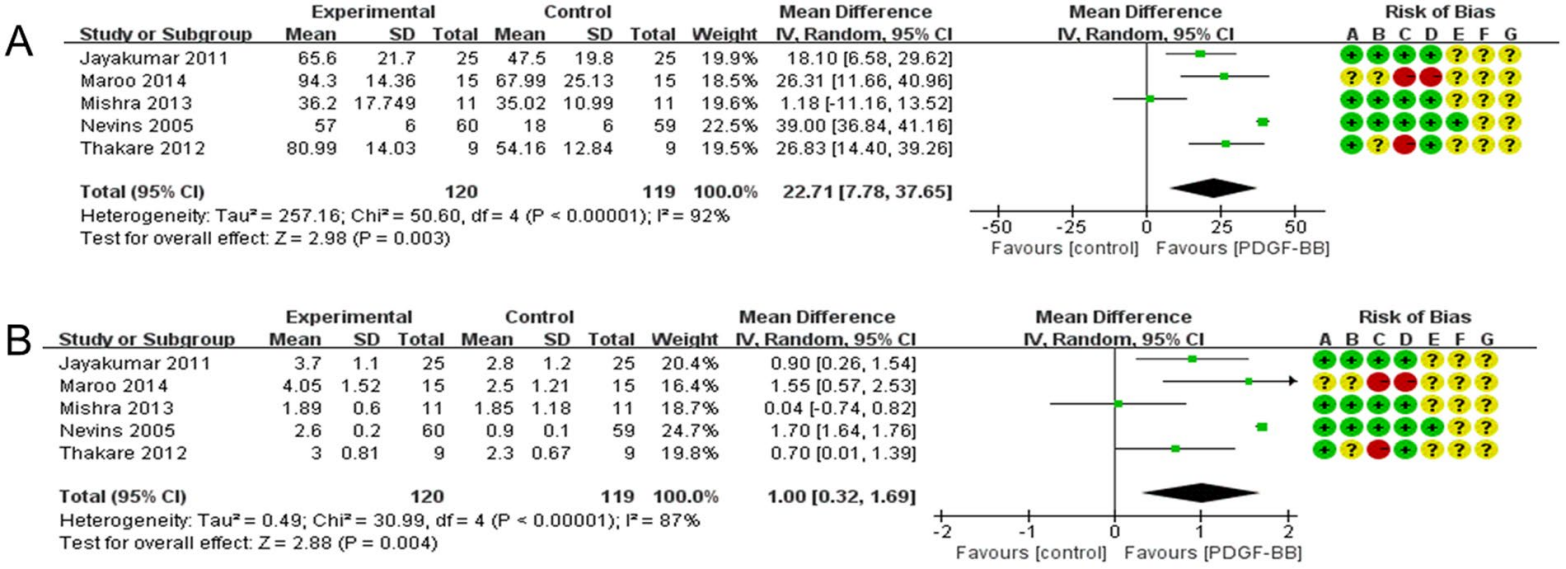
(**A**) Forest plot of comparison: 0.3 mg/ml PDGF-BB groups was compared with control groups among patients with osseous defects, outcome: BF%. (**B**) Forest plot of comparison: 0.3 mg/ml PDGF-BB was compared with control groups among patients with osseous defects, outcome: LBG.

We conducted a meta-analysis of five RCTs which reported researches on patients with osseous defects (Mishra^22^, Jayakumar^27^, Maroo and Murthy^28^, Nevins^33^, Thakare^36^; Fig. 3). Our analysis focused on the effect of rhPDGF-BB on BF% and LBG among these patients. The studies all utilized 0.3 mg/ml PDGF-BB in treatment groups. Mishra^22^ reported a BF% value of 36.2 ± 17.749 (N = 11) in the treatment group and 35.02 ± 10.99 (N = 11) in the control group. Research from Jayakumar^27^ reports a greater difference. The BF% value for the treatment group was 65.6 ± 21.7 (N = 25) and 47.5 ± 19.8 (N = 25) for the control group. Similarly Maroo and Murthy^28^ reported that the treatment group achieved a BF% of 94.3 ± 14.36 (N = 15), whereas the control figure was 67.99 ± 25.13 (N = 15). Nevins^33^ found an even greater difference. The BF% value for the rhPDGF-BB treatment group was 57 ± 6 (N = 60) and only 18 ± 6 (N = 59) in the control group. Thakare^36^ also explored the effect of rhPDGF-BB on patients’ BF%. The treatment group attained a BF% of 80.99 ± 14.03 (N = 9), as compared to the control group’s figure of 54.16 ± 12.84 (N = 9).

When the studies were pooled for meta-analysis, we found that the BF% of patients in the treatment groups, all of whom had received 0.3 mg/ml rhPDGF-BB, was 22.71% higher than that of patients in the control groups (MD = 22.71, 95%CI = 7.78~37.65, *p* = 0.003) (Fig. 3A). Our meta-analysis of LBG outcomes also showed significant differences in the predicted direction between treatment and control groups (MD = 1.00, 95%CI = 0.32~1.69, *p* = 0.004; Fig. 3B).

#### Secondary outcomes

The achievement of higher CAL-G ratings is a desirable secondary outcome of periodontal regeneration. We selected this variable as one of the clinical parameters in our meta-analysis of five RCTs (Mishra^22^, Jayakumar^27^, Maroo and Murthy^28^, Nevins^33^, Thakare^36^; S2. A). The CAL-G value for the treatment group in Mishra^22^ was 3 ± 0.89 mm (N = 11). The figure for the control group was 2.64 ± 067 mm (N = 11). Research from Jayakumar^27^ reported a similar difference in the same direction. The CAL-G value for the treatment group was 3.7 ± 1 mm (N = 27) and 2.8 ± 0.9 mm (N = 27) for the control group. Likewise, Maroo and Murthy^28^ reported that the CAL-G value for patients in the treatment group was 5.33 ± 1.72 mm (N = 15) whereas that of control group was 3.67 ± 1.45 mm (N = 15). Earlier research from Nevins^33^ had displayed a similar but weaker trend. The CAL-G value of treatment group patients was 3.8 ± 0.2 mm (N = 60). That of the control group was 3.5 ± 0.2 mm (N = 59). Similar results were found in Thakare^36^, whose treatment group patients showed a CAL-G of 3.42 ± 1.24 mm (N = 9), as distinct from the 2.06 ± 0.63 mm (N = 9) found among the controls.

Our meta-analysis of these studies, when pooled, showed a statistically significant mean CAL-G difference of 0.76 mm between the treatment and control groups in the predicted direction (MD = 0.76, 95%CI = 0.28~1.24, *p* = 0.002; S2. A). In addition our meta-analysis about PDR, which is another secondary outcome of periodontal repair, also indicated the positive impact of rhPDGF-BB treatment when compared to control groups (MD = 1.12, 95%CI = 0.28~1.96, *p* = 0.0001; S2. B; this finding is at odds with the conclusion drawn by Khoshkam^38^.).

#### The Effect of Rhpdgf-Bb on Patients with Gingival Recession

**Figure 4:**
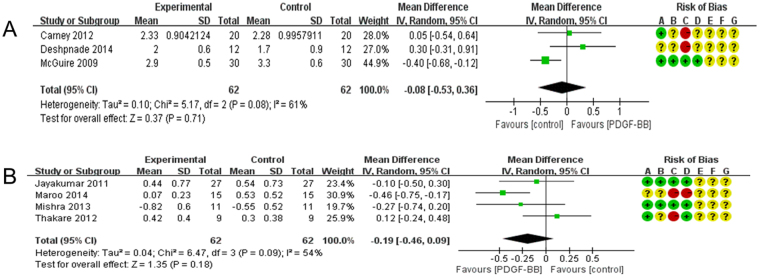
(**A**) Forest plot of comparison: 0.3 mg/ml PDGF-BB groups was compared with control groups among patients with gingival recession, outcome: GR. (**B**) Forest plot of comparison: 0.3 mg/ml PDGF-BB was compared with control groups among patients with osseous defects, outcome: GR.

We have also carried out a meta-analysis of the effects of 0.3 mg/ml rhPDGF-BB on patients whose principal problem is gingival recession, a periodontal symptom linked to soft tissue deficiency. We included three RCTs in our analysis (Carney^23^, Deshpnade^25^, McGuire^29^; Fig. 4A). The independent variable was the application of rhPDGF-BB to the treatment group. Our analysis treated the reduction of vertical GR as the principal dependent variable for assessing the effectiveness of this clinical intervention. Results were less clear in this matter. Carney^23^ reported that GR reduction was 2.33 ± 0.9042124 mm (N = 20) in the treatment group. In the control group the reduction figure was 2.28 ± 0.9957911 mm (N = 20). A similar trend emerged in Deshpnade^25^. The treatment patients experienced a reduction of 2 ± 0.6 mm (N = 12); the figure for the control patients was 1.7 ± 0.9 mm (N = 12). However, the findings in McGuire^29^ yielded the opposite trend. The treatment group had a reduction of 2.9 ± 0.5 mm (N = 30). The reduction in the control patients was slightly higher: 3.3 ± 0.6 mm (N = 30).

Our meta-analysis, however, found no statistically significant differences between the treatment and control groups on these variables (Fig. 4A). It can also be noted that the use of 0.3 mg/ml rhPDGF-BB produced no statistically significant effect on GR among patients suffering from periodontal osseous defect (Fig. 4B).

We also ran statistical tests on other clinical parameters to assess their impact on gingival recession. Among the variables examined were WKG, RCP, CAL-G and PDR. Our meta-analysis yielded no statistically significant difference in GR outcomes between the treatment groups receiving rhPDGF-BB and the control groups.

### Review of other Growth Factors in Periodontal Regeneration

Both Stavropoulos^39^ and Windisch^40^ studied the effectiveness of rhGDF-5 for periodontal regeneration, using data, however, from the same RCT. Their analyses differed in that one of them evaluated clinical and histological outcomes, whereas the other accessed clinical parameters and evidence concerning the safety of rhGDF-5. On the whole, both studies concluded that the use of rhGDF-5 was clinically safe and beneficial to periodontal repair. However, they found no statistically significant differences between treatment and control groups.

Howell^26^ reported a RCT that explored the impact of combining rhPDGF-BB and the insulin-like growth factor-I (IGF-I) on the treatment of periodontal diseases. It demonstrated that the combined treatment was safe but dose-dependent. The treatment with 150 mg/ml was significantly more effective in promoting bone generation than the use of a 50 mg/ml concentration.

## Discussion

Our meta-analysis warrants optimistic conclusions concerning the effectiveness of some of the treatments analyzed. For decades, the regeneration of periodontium, alveolar bone repair and the recuperation of periodontal soft tissue have been viewed as important long-term objectives in efforts to improve the prognosis for teeth with severe periodontal defects^1, 7^. However current periodontal treatment strategies usually rely on procedures targeting the junctional epithelium or on other interventions producing limited repair of damaged periodontium^4–7^. In recent decades the application of rhGFs to obtain periodontal regeneration has been proposed as a supplemental or alternative treatment strategy^10^. The proposal was initially based on several studies which measured the effect of periodontal repair in experiments on animals^8–14^. In addition to these animal experiments several clinical studies performed on humans had raised the possibility that rhPDGF-BB and rhFGF-2 were possibly beneficial for periodontal repair^13, 14, 22–36^. They thus emerged as promising clinical alternatives in regenerative periodontology.

In this regard, the two most commonly explored growth factors are rhPDGF-BB and rhFGF-2^10^. These have both been found to be actively involved in periodontal tissue renewal and the healing of wounds^10, 18^. This provided a physiological rational for considering their possible application to clinical situations.

Although two careful reviews had been published about the clinical efficacy of rhPDGF-BB and rhFGF-2 on periodontal healing, both of them suffered from some important weaknesses^37, 38^. Among the problems were: a limited number of RCTs, questionable or inconsistent inclusion and exclusion criteria, failure to distinguish among the growth factors as to their differential potential in periodontal repair, and failure to take into account the possible dose-dependent factors affecting their impact. These limitations motivated the undertaking of a more comprehensive and rigorous systematic review, based on meta-analysis procedures, concerning the efficacy of these two growth factors on periodontal repair. Our study suggests that both rhPDGF-BB and rhFGF-2 can effectively stimulate periodontal repair in a dose-dependent manner. It also finds, however, that the clinical efficacy of rhPDGF-BB among patients with osseous defects differs from its effect among those who suffer from gingival recession.

Certain methodological issues should be taken into account in future studies. The concentration-dependent effect of growth factors should be more carefully analyzed, as well as the differential effect of the application of growth factors to different patient populations. For example, we have noted that the growth factors operate differently in the healing of periodontal soft tissues and the healing of bone tissue.

The appearance of new relevant RCTs will call for an updating of the studies to be included in meta-analysis. The criteria for inclusion should also be improved. We encountered inconsistencies in the inclusion criteria utilized by Khoshkam 2015^38^ and Claudiu 2016^37, 38^. The meta-analysis of Khoshkam^38^ combined data from patients who had been treated with rhFGF-2 groups and those who had been treated with rhPDGF-BB^38^. In conducting their meta-analysis, they treated these different interventions as though they were the same. Their conclusion about the overall effect of GFs ignored the differential impact of these different GFs. Similarly, Claudiu 2016 merged rhPDGF-BB and rhGDF-5 patients into one treatment group, overlooking potential differences in the effects of these two GFs^37^. Furthermore, these studies were inconsistent in the criteria used for including or excluding RCTs from the meta-analysis. The study by Howell^26^ which combined PDGF-BB and IGF-1 patients into the same treatment group was included in the study of Khoshkam 2015^26, 38^. This weakened the validity of any conclusion, because two different interventions on the treatment group (rhPDGF-BB and rhIGF-1) were questionably treated as a single intervention. Also Claudiu 2016 probably erred by including Nevins^34^. This latter study is an extension of the earlier study reported in Nevins^33^. Six centers, however, had by then withdrawn reducing the number of patients whose clinical progress was measured^33, 34, 37^. In addition, the two studies phrased their meta-analysis in terms of the impact of rhPDGF-BB on patients with osseous defects. However, three of the RCTs included patients who received rhPDGF-BB for the treatment of gingival recession^23, 25, 29^. In view of potential differences in the therapeutic responses within these two different patient populations, it would appear necessary to carry out separate analyses for each.

It is worthwhile to examine this matter in some detail. The results of our study indicate that the use of 0.3 mg/ml rhPDGF-BB had a positive impact on BF%, LBG, CAL-G, and PDR among patients with osseous defects. However, when used in gingival recession treatment, rhPDGF-BB failed to achieve these effects. In such cases, this treatment had no statistically significant impact on patients’ GR, CAL-G, or PDR. This supports our hypothesis that the impact of rhPDGF-BB on patients suffering from osseous defect is different from its impact on those who suffer gingival recession. Hence, a conclusion can be drawn that 0.3 mg/ml rhPDGF-BB is a promising clinical candidate for stimulating the repair of periodontal osseous defect. But its efficacy in the repair of gingiva regeneration is still unproven.

The final and most important point concerns the issue of the most effective concentration of growth factors to use in clinical treatment. This issue was not examined in either Khoshkam^38^ or Claudiu 2016^37, 38^. Our meta-analysis, however, shows that the therapeutic impact of rhFGF-2 was dose-dependent. Specifically, 0.3% rhFGF-2 showed the greatest positive impact on BF% and LBG. In stark contrast, lower or higher concentrations (0.1% rhFGF-2 or 0.4% rhFGF-2) failed to induce statistically significant differences between treatment and control groups. More research is needed to replicate this counterintuitive finding. But it is clear that the concentration of rhFGF-2 affects its therapeutic efficacy. As to CAL-G, there was a positive (though not statistically significant) trend in which CAL-G increased along with an increase in the concentration of rhFGF-2. To sum up, rhFGF-2 is effective in stimulating BF% and LBG; its impact on CAL-G is less clear though promising; and the recommended concentration is 0.3%.

The effects of different concentrations of rhPDGF-BB were explored by both Nevins 2005 and Ridgway 2008^14, 33^. Unfortunately, the impact of dosage concentrations cannot be firmly established because Ridgway^14^ simply compared the impact of different dosages but did not establish control groups that received no growth factors^14^.

In conclusion, our findings will hopefully provide clinicians with guidelines for making appropriate choices with respect to the application of growth factors in clinical settings. It is also clear that more rigorously designed large scale research involving many centers will be required to confirm or modify the clinical guidelines that have been suggested by the results of this study.

## Methods

This meta-analysis was carried out in accordance with the guidelines in Preferred Reporting Items for Systematic Reviews and Meta-Analyses (PRISMA) and the Cochrane Handbook for Systematic Reviews of Interventions^41, 42^.

## Inclusion Criteria

### Types of studies

Both split mouth RCTs and parallel RCTs were included. Other study designs, such as case report, retrospective study, cohort study and so son, were excluded.

### Types of participants

The study included patients who had been diagnosed as having periodontal diseases based on the diagnostic guidelines of the American Academy of Periodontology (AAP)^43^.

### Types of interventions

Patients receiving the growth factors treatment were regarded as the intervention group, and the objective of the utilization of growth factors was to stimulate periodontal regeneration. The control group consisted of patients who did not receive growth factors treatment but who received conventional periodontal surgeries, placebo plus surgeries, or the carriers of growth factors plus surgeries.

### Types of outcome measures

#### Major dependent variables

The targeted outcome in this analysis was periodontal repair, or more precisely, evidence concerning the differential capacity of different growth factors to promote periodontal repair. New bone formation is a primary goal of clinical intervention for periodontal repair. In that light we chose an increase in BF% and LBG as the major measures of success in the repair of periodontal hard tissue. The choice of LBG as the dependent variable is particularly germane to evaluate the use of FGF-2 for patients with osseous defect. However, PDGF-BB was used on patients with gingival recession. In the case of these patients, the major dependent variable became GR.

## Exclusion Criteria

Published clinical trials were excluded if they did not meet the above inclusion criteria.

## Search Methods

A literature search was carried out within four databases: the Cochrane Central Register of Controlled Trials (CENTRAL; 2016), MEDLINE (via OVID, 1948 to August 2016), Embase (1984 to August 2015) and Pubmed (until August 2016). We also searched the online databases of the Journal of Periodontology, Journal of Clinical Periodontology, the Journal of Periodontal Research, International Journal of Periodontics & Restorative Dentistry, Periodontology 2000, Journal of Periodontal and Implant Science and the Journal of Dental Research. In addition, attention was paid to the references cited in journal articles. The search words consisted of both MeSH heading words and free text words. Among the latter were “periodontology”, “periodontal diseases”, “periodontitis”, “gingival recession”, “periodontal regeneration”, “periodontal repair”, “ gingivitis”, “clinical trials” and “randomized controlled trials”. These words were combined with synonyms for “growth factors”, “FGF”, “FGF-2”, “fibroblast growth factor”, “BMP”, “BMP-2”, “BMP-4”, “BMP-7”, “bone morphogenetic protein”, “TGF”, “transforming growth factor”, “PDGF”, “PDGF-BB”, “platelet-derived growth factor”, “IGF”, “IGF-1”, “insulin-like growth factors” “GDF” “GDF-5” or “growth differentiation factor”. The search was restricted to articles written in English.

## Study Inclusion

Three reviewers (LFF, YFY and XX) independently screened and evaluated the titles and abstracts which the preliminary search yielded. Subsequently the content of the articles that potentially met the inclusion criteria were examined. In the case of disagreement among the researchers, the decision to include a study or not in the meta-analysis was made only after the disagreements were resolved. If no consensus was reached by the investigators, an alternative investigator (LCJ) acted as an arbiter.

## Assessment of Risk of Bias

This evaluation was independently performed by two reviewers (LFF and YFY) with The Cochrane “risk of bias” instrument. Disagreements between estimators were resolved by discussion until consensus was reached.

## Data Extraction

Two independent estimators (LFF and YFY) extracted data from the studies that had been included. The data extracted included the following: the demographic profile of patients, the study design, the growth factors used as interventions, the method of control, the generation of randomization, methods of allocation to treatment groups and control groups, and procedures to ensure blindness. The most important data extracted, of course, were the measures of the results of the therapeutic interventions.

## Statistical Analysis

Statistical analyses were carried out utilizing Review Manager 5.1. Heterogeneity was assessed via the I^2^ statistic (a test for heterogeneity) on the level of α = 0.10. If there was considerable or substantial heterogeneity (I^2^ > 50%), a random-effects model was adopted; otherwise a fixed-effects model was used. The measures of the results of treatment were presented as mean difference (MD) utilizing 95% confidence intervals (CIs). Statistical significance was calculated at α = 0.05 (2-tailed z tests) and the threshold for statistical significance was set as *p* < 0.05. Besides, some studies did not provide the change values of certain outcomes and under such conditions the mean and standard deviation (SD) of change values were calculated according to a previously reported method and the Cochrane Handbook for Systematic Reviews of Interventions (Version 5.1.0) by a statistician(LCJ)^44^. The mean and SD values in the baseline and follow-up endpoints were extracted by the estimators mentioned above. In this study, the change values were included into quantitative study based on the value of Corr is 0.5 according to previous study^44^.

## Electronic supplementary material

Supplemental Data
