# Supplementary material for: Evaluation of Recombinant Human FGF-2 and PDGF-BB in Periodontal Regeneration: A Systematic Review and Meta-Analysis
Source: Sci Rep. 2017 Mar 6;7:65. doi: 10.1038/s41598-017-00113-y (PMC5427916; doi:10.1038/s41598-017-00113-y)
Supplement: Supplementary file 1 — Supplemental Data [file 41598_2017_113_MOESM1_ESM.pdf]

Evaluation of Recombinant Human FGF-2 and PDGF-BB in Periodontal Regeneration: A Systematic Review and Meta-Analysis

Li Feifei[1,2*], Yu Fanyuan[1,2*], Xu Xin[1,2], Li Chunjie[1,2], Huang Dingming[1,2], Zhou Xuedong[1,2], Ye Ling[1,2#], Zheng Liwei[1,2#]

1. State Key Laboratory of Oral Diseases, National Clinical Research Center for Oral Diseases, West China Hospital of Stomatology, Sichuan University, Chengdu, China
2. West China School of Stomatology, Sichuan University, Chengdu, China

* Li Feifei, Yu Fanyuan contributed equally to this work.
# Corresponding authors: Zheng Liwei and Ye Ling

Zheng Liwei: DDS, PhD, State Key Laboratory of Oral Diseases, National Clinical Research Center for Oral Diseases, West China Hospital of Stomatology, Sichuan University, No.14, Section 3, South Renmin Road, Chengdu, China, 610041.
Telephone: 862885503469
Fax: 862885582167
E-mail: liwei.zheng@scu.edu.cn

Ye Ling: DDS, PhD, Department of Endodontics, West China Hospital of Stomatology, Sichuan University, No.14, Section 3, South Renmin Road, Chengdu, China, 610041.
Telephone: 862885503497
Fax: 862885582167
E-mail: yeling@scu.edu.cn

Supplemental Data

S1

A

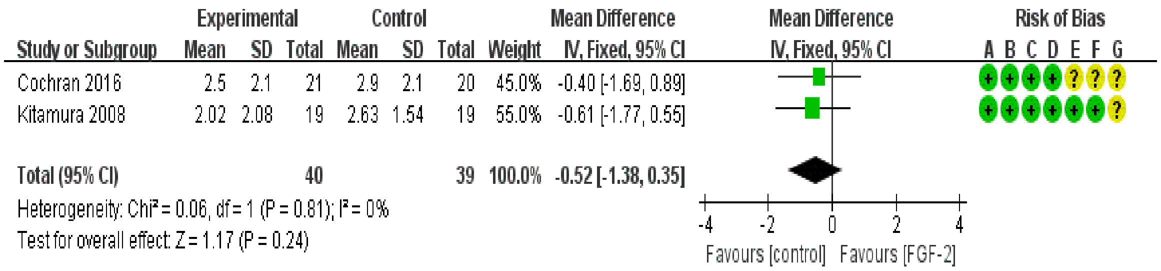

B

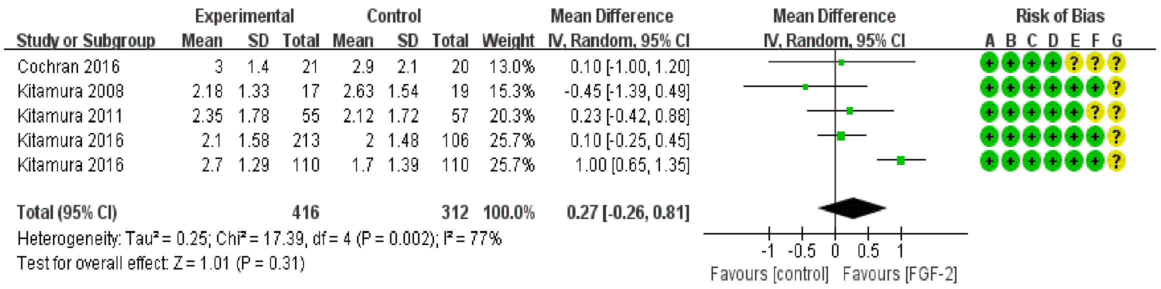

C

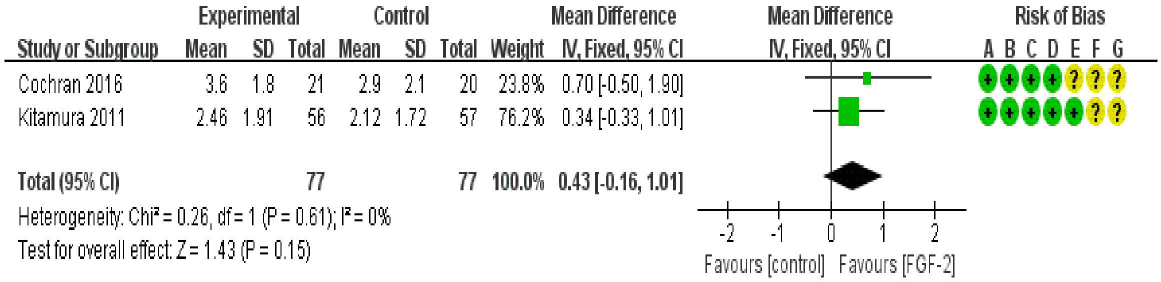

S1A. Forest plot of comparison: 0.1% FGF2 groups was compared with control groups among patients with osseous defects, outcome: CAL-G.

S1B. Forest plot of comparison: 0.3% FGF2 groups was compared with control groups among patients with osseous defects, outcome: CAL-G.

S1C. Forest plot of comparison: 0.4% FGF2 groups was compared with control groups among patients with osseous defects, outcome: CAL-G.

S2

A

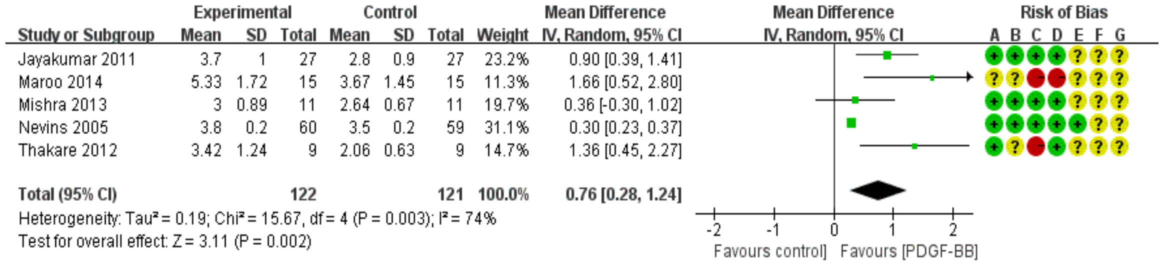

B

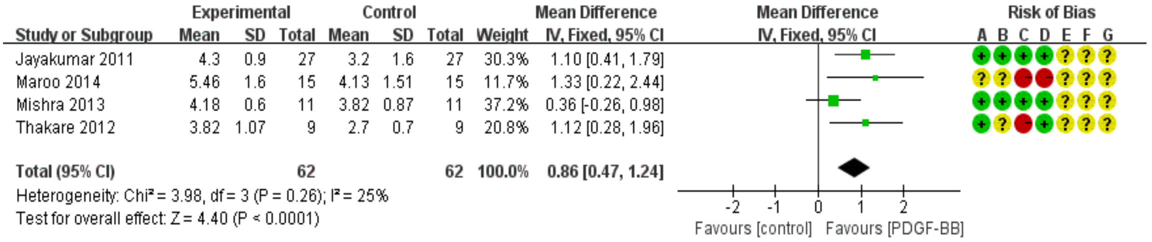

S2A. Forest plot of comparison: 0.3mg/ml PDGF-BB groups was compared with control groups among patients with osseous defects, outcome: CAL-G.
S2B. Forest plot of comparison: 0.3mg/ml PDGF-BB groups was compared with control groups among patients with osseous defects, outcome: PDR.

S3

A

Study or Subgroup	Experimental Mean	Experimental SD	Experimental Total	Control Mean	Control SD	Control Total	Weight	Mean Difference IV, Fixed, 95% CI
Carney 2012	2.06	1.175117	20	2.4	1.05532	20	22.3%	-0.34 [-1.03, 0.35]
Deshpnade 2014	1.9	1.3	12	1.5	0.6	12	16.3%	0.40 [-0.41, 1.21]
McGuire 2009	2.9	0.6	30	2.9	1	30	61.4%	0.00 [-0.42, 0.42]
**Total (95% CI)**			**62**			**62**	**100.0%**	**-0.01 [-0.34, 0.32]**

Heterogeneity: Chi² = 1.86, df = 2 (P = 0.39); I² = 0%
Test for overall effect: Z = 0.06 (P = 0.95)

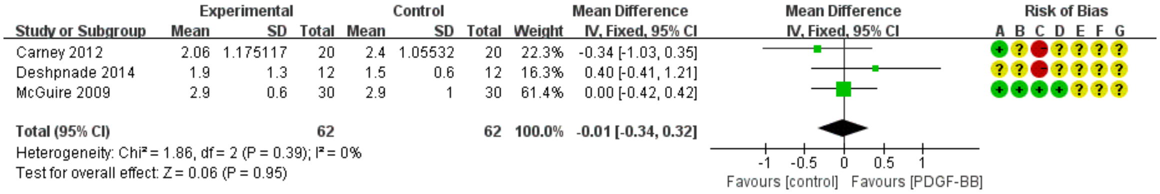

B

Study or Subgroup	Experimental Mean	Experimental SD	Experimental Total	Control Mean	Control SD	Control Total	Weight	Mean Difference IV, Fixed, 95% CI
Carney 2012	0.09	0.3858756	20	0.25	0.35	20	59.7%	-0.16 [-0.39, 0.07]
Deshpnade 2014	0.4	0.7	12	0.5	1	12	6.5%	-0.10 [-0.79, 0.59]
McGuire 2009	0	0.6	30	0.4	0.6	30	33.8%	-0.40 [-0.70, -0.10]
**Total (95% CI)**			**62**			**62**	**100.0%**	**-0.24 [-0.41, -0.06]**

Heterogeneity: Chi² = 1.70, df = 2 (P = 0.43); I² = 0%
Test for overall effect: Z = 2.63 (P = 0.008)

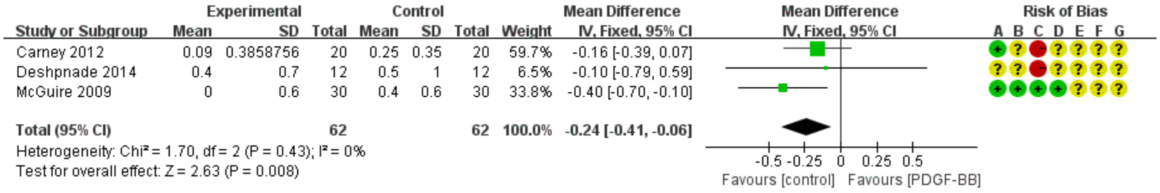

C

Study or Subgroup	Experimental Mean	Experimental SD	Experimental Total	Control Mean	Control SD	Control Total	Weight	Mean Difference IV, Fixed, 95% CI
Carney 2012	0	1.15	20	-0.14	1.04	20	0.5%	0.14 [-0.54, 0.82]
Deshpnade 2014	0.8	1	12	1.5	1.2	12	0.3%	-0.70 [-1.58, 0.18]
McGuire 2009	1	0.1	30	1.3	0.1	30	99.1%	-0.30 [-0.35, -0.25]
**Total (95% CI)**			**62**			**62**	**100.0%**	**-0.30 [-0.35, -0.25]**

Heterogeneity: Chi² = 2.40, df = 2 (P = 0.30); I² = 17%
Test for overall effect: Z = 11.63 (P < 0.00001)

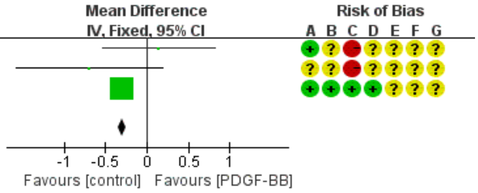

D

Study or Subgroup	Experimental Mean	Experimental SD	Experimental Total	Control Mean	Control SD	Control Total	Weight	Mean Difference IV, Random, 95% CI
Deshpnade 2014	87.7	12.9	12	68.6	25.7	12	45.3%	19.10 [2.83, 35.37]
McGuire 2009	90.8	2.8	30	98.6	1	30	54.7%	-7.80 [-8.86, -6.74]
**Total (95% CI)**			**42**			**42**	**100.0%**	**4.37 [-21.87, 30.62]**

Heterogeneity: Tau² = 327.20; Chi² = 10.46, df = 1 (P = 0.001); I² = 90%
Test for overall effect: Z = 0.33 (P = 0.74)

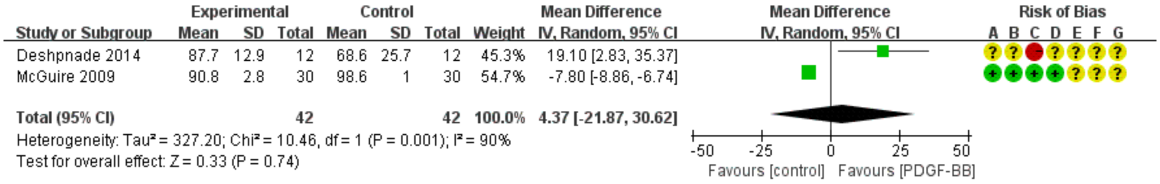

S3A. Forest plot of comparison: 0.3mg/ml PDGF-BB groups was compared with control groups among patients with gingival recession, outcome: CAL-G.

S3B. Forest plot of comparison: 0.3mg/ml PDGF-BB groups was compared with control groups among patients with gingival recession, outcome: PDR.

S3C. Forest plot of comparison: 0.3mg/ml PDGF-BB groups was compared with control groups among patients with gingival recession, outcome: WKT

S3D. Forest plot of comparison: 0.3mg/ml PDGF-BB groups was compared with control groups among patients with gingival recession, outcome: PRC..
